# Waiting upon the Remedy

**Published:** 1894-12

**Authors:** F. Preston

**Affiliations:** Chester


### WAITING UPON THE REMEDY.

CHESTER, Nov. 17th, 1894.

EDITOR HOMŒOPATHIC PHYSICIAN:—I wish to call your attention to the annexed article, on account of its one peculiarity. We are here shown the very unusual spectacle of a physician of the dominant school waiting upon the action of the remedy, viz.: Antitoxin.

Antitoxin is now on trial in the United States, and the appended article is a fair sample of how the trial is being conducted. The writer of this Antitoxin trial seems convinced of the "wonderful abortive power" of the new remedy. He continued his treatment of the case by other methods, after having injected Antitoxin, and then tells us of the "wonderful abortive power" of the new remedy! Thirty-six hours elapse while he is waiting on the action of the medicine administered hypodermically; a rather powerful remedy being continually applied to the diseased surface by means of a spray at same time.

This is puerile: It is like the child blowing wind into the sails of his toy-boat, floating in a basin, and imagining a gale. But, so far as the gentleman's capability went, he really "waited on the action of the remedy." In fine, he did not fly into a passion with his Antitoxin; but, without sight of a "reaction" (that "reaction" so dear to the bacteriological heart, and of which we read so much in the closing days of the Tuberculin boom), he waited thirty-eight hours, there or thereabouts, and the patient recovered. He goes on record as the pioneer waiter-on-the-action-of-the-remedy of the dominant school.

Now let us suppose that we had administered Lach.$^{cm}$ or the Diphtherinum of the late lamented Dr. Samuel Swan, how many adjuvants do you suppose would have been tolerated by our critics, or asked for by us?

Antitoxin is on trial, and Dr. Arnold W. Catlin, of Brooklyn, has shown us how.

I have cured cases of diphtheria, in my day and generation, when I waited on the indicated remedy. If I failed or fail to wait they die. I fear they will die occasionally under Antitoxin, and, as Hering used to say, "and some others." But, though our friends are still using the "omnibus" prescription, one of them waited thirty-six hours. He, at least, had heard whispered somewhere the proverb :

"Tout vient à point pour que sait attendre."

Very truly yours,
F. PRESTON.

